# Trauma Ultrasound in Civilian Tactical Medicine

**DOI:** 10.1155/2012/781570

**Published:** 2012-11-29

**Authors:** Lori Whelan, William Justice, Jeffrey M. Goodloe, Jeff D. Dixon, Stephen H. Thomas

**Affiliations:** Department of Emergency Medicine, University of Oklahoma School of Community Medicine, Tulsa, OK 74104, USA

## Abstract

The term “tactical medicine” can be defined in more than one way, but in the nonmilitary setting the term tactical emergency medical services (TEMS) is often used to denote medical support operations for law enforcement. In supporting operations involving groups such as special weapons and tactics (SWAT) teams, TEMS entail executing triage, diagnosis, stabilization, and evacuation decision-making in challenging settings. Ultrasound, now well entrenched as a part of trauma evaluation in the hospital setting, has been investigated in the prehospital arena and may have utility in TEMS. This paper addresses potential use of US in the tactical environment, with emphasis on the lessons of recent years' literature. Possible uses of US are discussed, in terms of both specific clinical applications and also with respect to informing triage and related decision making.

## 1. Introduction

The use of ultrasound (US) outside the radiology suite has grown markedly over the past few decades [1]. With increasing portability of US machines has come more frequent utilization of the technology at the “point of care” site. 

Over a decade ago, investigators began assessing deployment of US for use even before patients arrived at the hospital [2]. First for patients in trauma arrest, and then for general trauma diagnosis, the US was shown to have utility in the field [3]. While initial applications of US were somewhat limited by technical difficulties, the passage of time saw improvements translating into an ability for prehospital providers to regularly obtain interpretable US images [4].

Uses of US in the hospital and ambulance EMS settings have been well reviewed elsewhere [1, 4, 5]. There is less evidence in the realm of special operations support, such as that provided for special weapons and tactics (SWAT) team and similar law enforcement operations. A term used to denote the medical support for law enforcement operations, as provided by physician or nonphysician prehospital personnel, is “tactical emergency medicine support” or “tactical emergency medical services” (TEMS). 

 The goal of this paper is to overview the potential uses of US for trauma evaluation and care in the TEMS environment. The paper includes performance of US by physicians and nonphysicians and addresses specific clinical situations in which US could potentially be helpful in tactical medicine. The paper's intent is not to be comprehensively inclusive of all relevant literature; the aim is rather to address specific clinical situations and provide illustrative references from the past five years' literature.

Many uses of US have been investigated and reported in the prehospital setting. Some of these, such as cardiac output estimation or differentiation of chronic obstructive pulmonary disease (COPD) from congestive heart failure (CHF), are promising but not likely to be applicable in most tactical settings [6–8]. These types of US uses may occasionally have applicability, especially in disaster or mass casualty situations or in austere environments [9], but these and similar nontrauma US applications will not be discussed here. 

With the understanding that there may be other situations in which US could be of aid, this paper includes discussions of US use for triage, for trauma diagnostic applications (for cardiac, pulmonary, vascular, abdominal, retinal, and extremity imaging), and as an aid for evacuation decision-making in TEMS. The discussion does not extend to scientific or technical information about US, available machines, or how to obtain or interpret images. Readers are referred to standard texts and related sources, for this information.

The first applications covered are for cardiac imaging or echocardiography. Lung-related US is discussed next, followed by inferior vena cava (IVC) and abdominal imaging. Extremity applications, including both fracture and vascular assessment, are covered next. The final clinical situation considered is retinal scanning to detect increased intracranial pressure (ICP). The paper's final sections address triage, feasibility, and areas for further research in TEMS application of US. It is hoped that the information in the paper can be of utility to TEMS programs considering US investigations, deployment, and/or educational efforts. 

## 2. Cardiac Ultrasound

Echocardiography is one of the oldest and best-characterized uses of US in the hospital, with many anatomical and functional applications [1]. On a much more limited basis, the ability to perform cardiac imaging in the TEMS setting offers multiple benefits. Given concerns about image quality and the level of training required for advanced cardiac US interpretation, the primary areas of US potential for TEMS are identification of cardiac motion and diagnosis of pericardial effusion. 

### 2.1. Cardiac Standstill

In the Emergency Department (ED) or even in routine EMS settings, the timely identification of patients in whom resuscitation should be halted is usually not considered a major challenge. There is little risk to inadvertently prolonging resuscitation efforts. In tactical medical situations, though, extraction and evacuation can occasionally pose significant risk to law enforcement members (operators) and/or medical team members. Furthermore, if there are multiple casualties, early determination of which patients are nonsalvageable can help focus resources—resources that are much more seriously limited in TEMS settings than in the ED. 

For the reasons just iterated, the use of tactical medicine echocardiography for assessment for cardiac motion is attractive. For those patients who can be clearly identified to have cardiac standstill after trauma, US can allow for conservation of air or ground evacuation resources and eliminate risks attendant to extracting and transferring patients with no chance of survival. Prehospital physicians have been shown to be able to reliably identify cases of cardiac standstill (and thus predict who will fail to survive if transported to the ED) [10, 11]. Whether images are read by forward medical care providers or transmitted (see discussion below) for interpretation by physicians in the rear echelons (or in the ED), tactical echocardiography is a potentially useful tool for determination of whether resuscitation efforts are futile. 

### 2.2. Pericardial Tamponade

 Most TEMS injuries are penetrating, and therefore traumatic pericardial effusion is a possible diagnostic entity in cases of chest injury. It is well known that US can diagnose traumatic pericardial effusion when deployed in the prehospital (standard EMS) setting [12]. On occasion, prehospital US has not only detected pericardial tamponade, but also has guided successful treatment in the setting of penetrating trauma [13]. In the special operations setting, where patients may be hours away from definitive care, there is thus particular attractiveness to the possible deployment of portable US to definitively identify the presence (or absence) of pericardial effusion and tamponade. Given the frequency of penetrating trauma, and the likelihood of reversibility of pericardial tamponade with appropriate treatment (even if solely temporizing), TEMS medical care is likely to benefit from the use of US for this indication.

## 3. Pulmonary Ultrasound

One of the most important uses of US for trauma is in the rapid identification of pneumothorax [1]. Since pneumothorax is one of the major life risks in the penetrating trauma mechanisms that characterize most TEMS situations, US of the lungs takes on major importance. Additional uses of US that are based upon the same signs (e.g., the “sliding lung sign” or SLS) used to assess for pneumothorax, include its application to assess endotracheal tube (ETT) positioning. As a less important but occasionally useful application, US may also be used to guide (halt) fluid resuscitation by identifying pulmonary congestion.

### 3.1. Pneumothorax

Pneumothorax is a major, yet treatable, cause of death in penetrating trauma. Prehospital identification of pneumothorax with US has already been successfully taught, in a French system that emphasized the importance of identifying pneumothorax in the field to enable immediately life-saving interventions [14]. There are *caveats* regarding application of US (e.g., need for a lateral view to avoid underdiagnosis) [15], but existing evidence supports pursuing both physician and nonphysician use of US to identify pneumothorax in the field [16].

In the TEMS situation, unnecessary needle thoracostomy can remove a needed law enforcement officer from tactical utility, and also may necessitate both evacuation and invasive follow-up procedures. Thus, in tactical medicine one of the most important uses of US for evaluation of the pleural space is the potential ability of the imaging to rule out pneumothorax and thus avoid nonindicated interventions. Prehospital US has been identified as an important tool to prevent placement of unnecessary needle thoracostomies and also reduce requirements for resource-intensive evacuation [17].

When needle thoracostomy has been performed, US can be used to assess the effectiveness of the procedure. US has been recommended as a screening tool for patients who are status post needle thoracostomy, to determine if they really need tube thoracostomy in the ED [18, 19]. These recommendations may be extrapolated to TEMS utility of US to assess not only the need for, but also the effectiveness of, needle thoracostomy in the tactical setting. Given the muscular body habitus of many law enforcement officer “patients” and the potential inexperience of those performing TEMS needle thoracostomies, there is reason to desire a quick and accurate mechanism for confirming procedural success. 

### 3.2. Endotracheal Tube Positioning

The placement of an ETT is a relatively infrequent occurrence in TEMS, but the confirmation of intratracheal placement is of course a priority in those cases where intubation is performed. Similarly, rescue airways such as laryngeal mask airways also need to be evaluated for their functionality in ventilating patients. Traditional mechanisms for ongoing evaluation of ETT placement (e.g., capnography) may be scarce or difficult to deploy in the TEMS setting.

Fortunately, some of the standard imaging techniques that can be used to assess for pneumothorax are also useful for indirect confirmation of bilateral ventilation. Specifically, if the SLS is visualized on both sides of the thorax concomitant with inspiration there is a strong suggestion that both lungs are being ventilated; US can both detect endotracheal positioning and also mainstem intubation [20]. This has been demonstrated to be useful even in the austere settings where other means of ETT placement confirmation are lacking [21].

### 3.3. Fluid Status

The assessment of volume status for CHF identification has been demonstrated to be useful, even in challenging environments [9]. Acute CHF due to standard cardiac causes, is an unlikely finding in the TEMS setting. However, TEMS sonographers could find US useful as a guide in any situations in which there are questions of fluid overload from overzealous fluid administration as part of initial trauma resuscitation efforts. US identification of classic findings (i.e., multiple sonographic B-lines) can allow for early identification of fluid overload and pulmonary congestion, thus directing changes in field patient management [9]. This is potentially an area of utility for US in TEMS, especially in cases in which evacuation is delayed, and care in the tactical environment must last for a long time. 

## 4. Inferior Vena Cava Ultrasound and Volume Status

 While pulmonary US can demonstrate volume overload by demonstrating the presence of congestion and vascular overload, imaging of the IVC can be very useful at the other (low) end of the volume status spectrum. The technique has been rarely used in the out-of-hospital setting, but ED practitioners and others have reported utility to IVC imaging (e.g., during the respiratory cycle) as a reliable indicator of volume status, and therefore as a guide to fluid resuscitation [1, 22]. For the TEMS setting, in which there may be more restricted access to resuscitation fluids (which are heavy to carry), early demonstration of adequate fluid status can volume replacement resources.

## 5. Abdominal Injury

Identification of free fluid in the peritoneal space is a well-demonstrated use of trauma US, including in the prehospital setting where its application can lead to up-triaging patients and arrangements for expedited transport to the operating suite [23, 24]. Studies have suggested that both helicopter and ground EMS providers (with training levels down to the level of EMT-Intermediates) can obtain and interpret abdominal images for trauma diagnosis [23]. 

The primary utility in field US assessment for intraabdominal fluid appears to be triage and evacuation prioritization. Hypotension in the setting of free fluid on US could also guide administration of field resources such as tranexaminic acid. Given the state of the evidence, it is reasonable to include search for peritoneal fluid in the protocol for special operations sonography training, with the *caveat* that (as is the case for essentially all TEMS US use) further data are needed before final adoption of a protocol.

## 6. Fracture Diagnosis

Detection of fractures in the special operations setting is quite important. The identification of a fracture (or confirmation of its absence) can guide important triage and evacuation decisions. Thus, US results can potentially inform decisions such as whether to plan on returning a team member back to mission or order evacuation using scarce vehicular resources. 

Overall accuracy of US for fracture determination in austere (military) environments has been found to be very high [25]. Other studies have suggested similarly high accuracy of US for fracture diagnosis in the wilderness medicine setting (which in many ways approximates the TEMS environment) [26, 27].

Just as US can be useful to diagnose fractures, postreduction images can be used to assess bony alignment before splinting or evacuation. Furthermore, US can be used to assess arterial pulses distal to an injury site, both before and after any reduction or splinting (see Section 7).

## 7. Extremity Vascular Function

 When decisions about evacuation are on the line, the capability to assess vascular function distal to an extremity injury can be critical. Fractures that are stabilized and splinted do not necessarily warrant emergent evacuation, but such evacuation is indeed necessary if there is distal lack of perfusion. Doppler US can also be used to assess functionality of tourniquets, and this has been shown effective as a measuring tool in multiple studies [28, 29]. 

Documentation of a pulse in the radial or femoral region is useful as a gross estimation of blood pressure [30]. Since palpation of pulses in tactical situations can be challenging for a number of reasons (e.g., noise, patient positioning), the ability to easily assess for pulses with US can be of potential utility.

## 8. Intracranial Pressure 

Retinal US is a useful tool for detection of elevated ICP in the setting of trauma [1, 31]. While there has been little or no investigation of this application of US in the TEMS setting, the early diagnosis of elevated ICP (i.e., demonstration of increased optic nerve sheath diameter) could have particular utility in tactical medicine. Triage decisions as well as evacuation modality decisions (e.g., air versus ground) could be informed by either reassuring findings or concerning indicators on ocular US.

## 9. Triage

While many TEMS operations occur in urban settings in which evacuation is not problematic, there are also cases in which evacuation of casualties is logistically challenging, dangerous, or both. In these situations, the ability to accurately triage patients and allocate scarce resources is not just a matter of overloading the regional trauma center—it is potentially a matter of life and death for the injured patients or for others who would either participate in extraction or potentially need resources that should not be wasted on trivially injured cases. US has been demonstrated to be occasionally useful in triage, both in the trauma arrest situation and also as a part of the overall evaluation of injured patients [2, 3]. 

### 9.1. Identification of the Dead

As previously noted, identification of cardiac standstill and lack of valvular motion is a reliable mechanism to demonstrate death in the field. This end of the triage scale is important as a possible area for US contributions to decision-making algorithms involving evacuation in austere and potentially dangerous settings.

### 9.2. Triage of Intermediate-Acuity Patients

In the “survivable injury” end of the spectrum, US application can differentiate patients who need immediate evacuation for life- or limb-saving care, from those who can be evacuated by ground or even be cleared for return to mission. US has been explicitly considered as a mechanism to improve trauma triage for those intermediate-acuity patients who may need helicopter transport [24]. In addition to ruling out serious injury, US can reduce undertriage [32].

## 10. Feasibility of Portable US Use in TEMS

If US is insufficiently rugged or otherwise unsuited for use in the out-of-hospital setting, the preceding sections' potential utilities for the technology are moot. The question is relevant, given results from some early studies of US in the prehospital setting. A 2001 publication, for instance, reported that FAST exams could not be performed in nearly half of patients due to time or patient access or equipment restrictions; those air medical researchers understandably concluded that US had a long way to go before it was feasible for field use [33]. At that time, technical difficulties (e.g., screen visualization, battery failure, and machine malfunction) prevented US image acquisition in one in five cases in which US was attempted [33].

More recent evidence strongly supports the notion that US has overcome its initial deployment difficulties. As US engineering has evolved, it has been demonstrated that US can be performed in a variety of prehospital settings, including stationary and moving vehicles [34]. Furthermore, image quality is good. Nonphysician EMTs have been successfully trained to execute prehospital US to assess for pneumothorax, pericardial effusion, and presence/absence of cardiac activity. For instance, after completing just one 2-hour course, EMTs in a San Antonio pilot study were able to obtain and interpret US images [35]. Other investigators have reported similar successes, with course durations (for physicians and paramedics) ranging from a half-day to a full day being found adequate to teach both image acquisition and interpretation [36, 37].

Feasibility of TEMS US use is suggested by promising results from studies that concluded various sonographic signs can be easily taught to prehospital providers. For example, the SLS is demonstrated to be relatively simple to teach when the goals are limited to interpretation for the presence of pneumothorax or effective ventilation (as previously discussed) [38]. Prehospital providers at the nonphysician level have been found to be able to learn the SLS in a cadaveric model, and also to retain the information when retested 9 months after initial training [16]. These educational data are obviously not conclusive, but they do provide a basis to justify further pursuit of educating TEMS practitioners on use of the technology.

Another angle on US is the potential for data acquisition to occur “on-site” (at the patient) and then be transmitted for remote interpretation. One group has found that paramedics with minimal US training were able to obtain and transmit readable trauma US images; the image obtaining and interpretations were done by physicians in another room [39]. Even more promising—and fertile ground for TEMS US research—is the concept of longer-range image transmission. This image transmission could be effected by either cellphone or wireless technology; the latter has already been demonstrated feasible in a U.S. Army study of both still and moving images [40].

 The question of feasibility of US in TEMS has not been definitively demonstrated. Expert reviewers considering the paramedic application of US judged the literature has “demonstrated that with the right education and mentorship, some paramedic groups are able to obtain US images of sufficient quality to positively identify catastrophic pathologies.” The reviewers concluded that “more research is required to demonstrate that these findings are transferable” to EMS, and further study is also needed to determine how prehospital US can facilitate improved patient outcomes [4]. Other reviewers have concurred that more research is needed, and that there is no clear link between prehospital US use and improved trauma outcomes [41]. 

Despite the opinions of skeptics, the bulk of the evidence is, in the opinion of this discussion's authors, supportive of a move towards further assessment of the technology. In terms of both personnel (e.g., education) and machine (e.g., image quality), the foundation for further exploration of US in TEMS has been laid. Recent review articles have noted portable US use for prehospital trauma evaluation and triage, assessment for pneumothorax, and other applications, with a wide range of outcomes (e.g., alterations in medical care, increases in diagnostic accuracy) [42]. 

Promise for US in the TEMS environment also comes from a 2012 review of the evidence (consisting of five studies) addressing US use in the military special forces setting [43]. These studies reported positive results with special forces nonphysicians' use of US for presence/absence of cardiac activity, pneumothorax evaluation, and fracture assessment. The reviewers concluded that, even in the austere conditions of special operations, nonphysician “medics can perform US with a high degree of accuracy.” The reviewers, noting there were few data addressing long-term recall of US training, call for continued analysis of US in the out-of-hospital setting and “exploration of the optimal curriculum to introduce this skill” [43]. 

## 11. Next Steps and Further Research

Important next steps for US research in TEMS include multiple areas for focus. Among the most important are (1) testing of the hypothesis that demonstrations of US feasibility in the “standard EMS” setting translate into TEMS functionality, (2) evaluation of which US clinical indications are most important and most viable for TEMS use, and (3) development and rigorous assessment of educational models for initial and ongoing training. 

The first of the three iterated areas for research deals with subjects ranging from image acquisition to transmission of US data for real-time assistance with interpretation. The second general area of investigation would include both assessment of the potential clinical uses already mentioned, and perhaps study of other areas for which TEMS US could be useful. These additional areas, including for instance skull fracture diagnosis [44, 45] and guidance of prehospital nerve blocks [46, 47], may have limited application in tactical medicine, but they appear worth consideration. Furthermore, it is likely that as TEMS operators begin to use US more, further unforeseen indications and applications may be identified (as occurred when US was introduced into the ED setting). Finally, education will need to be developed that builds on excellent programs already developed for nonradiologist US use, while incorporating the TEMS aspects with regard to personnel, equipment, and environment.

## 12. Conclusions

For most indications, data directly addressing US use in the TEMS setting are sparse, if present at all. Extrapolation of information from other settings, either from the ED or even the “standard” EMS arena, risks error. However, the existing evidence does support a potential role for US in TEMS. In terms of assisting diagnosis and medical management, as well as informing triage and resource utilization and personnel evacuation decision-making, US appears to have major potential. Due to the seemingly significant potential for US in TEMS, and the current lack of applicable data, focused research on clinical application of US in tactical medicine is warranted. 
